# Euregional Health Atlas: how to use it for cross-border public health policy Building a common health monitoring platform for the Meuse-Rhine Euroregion

**DOI:** 10.1093/eurpub/ckac129.216

**Published:** 2022-10-25

**Authors:** C Stabourlos

**Affiliations:** Service Lifestyle and Chronic Diseases Team, Sciensano, Brussels, Belgium

## Abstract

Studies show that European border regions -that account for over 30% of all European citizens- are faced with more deficits. Living in a border region still means fewer possibilities in the fields of employment, mobility, care and well-being. In order to tackle this, an absolute prerequisite is to dispose of relevant and comparable data across borders. The Euregional Health Atlas is an initiative that aims to collect and to visualize validated and comparable data for the municipalities of the Euregio Meuse-Rhine (EMR: Zuid Limburg (Nl), Zweckverband Aachen (De), the provinces Limburg, Liège, Ostbelgien (Be)). Themes addressed are: demographics (population, socioeconomic status, vulnerable groups), health care (care contacts, difficulties in daily living, chronic diseases, mortality), lifestyle (use of alcohol, smoking, weight, nutrition) and quality of life (perceived health, happiness).

Data used to feed the Euregional Health Atlas are derived from registers, health surveys and from former and current Interreg projects like the Youth Euregional Scan (YES). During the COVID-19 pandemic, data on the daily number of cases and 7-day incidence, the number of tests performed, the hospitalisation rate and the number of reported deaths related to Covid-19 were processed and presented on our dashboard and this not only for the Euregio Meuse-Rhine, but also for other Euregios. The Euregional Health Atlas is a work in progress: existing data are continuously being updated to improve their suitability for comparison purposes and new data are added to broaden the perspective. It contributes to knowledge-sharing, mutual understanding and cross-border policy development. The Atlas is a free, easily accessible platform, available for everybody; health care professionals, policy makers and citizens, see www.euregionalhealthatlas.eu!

